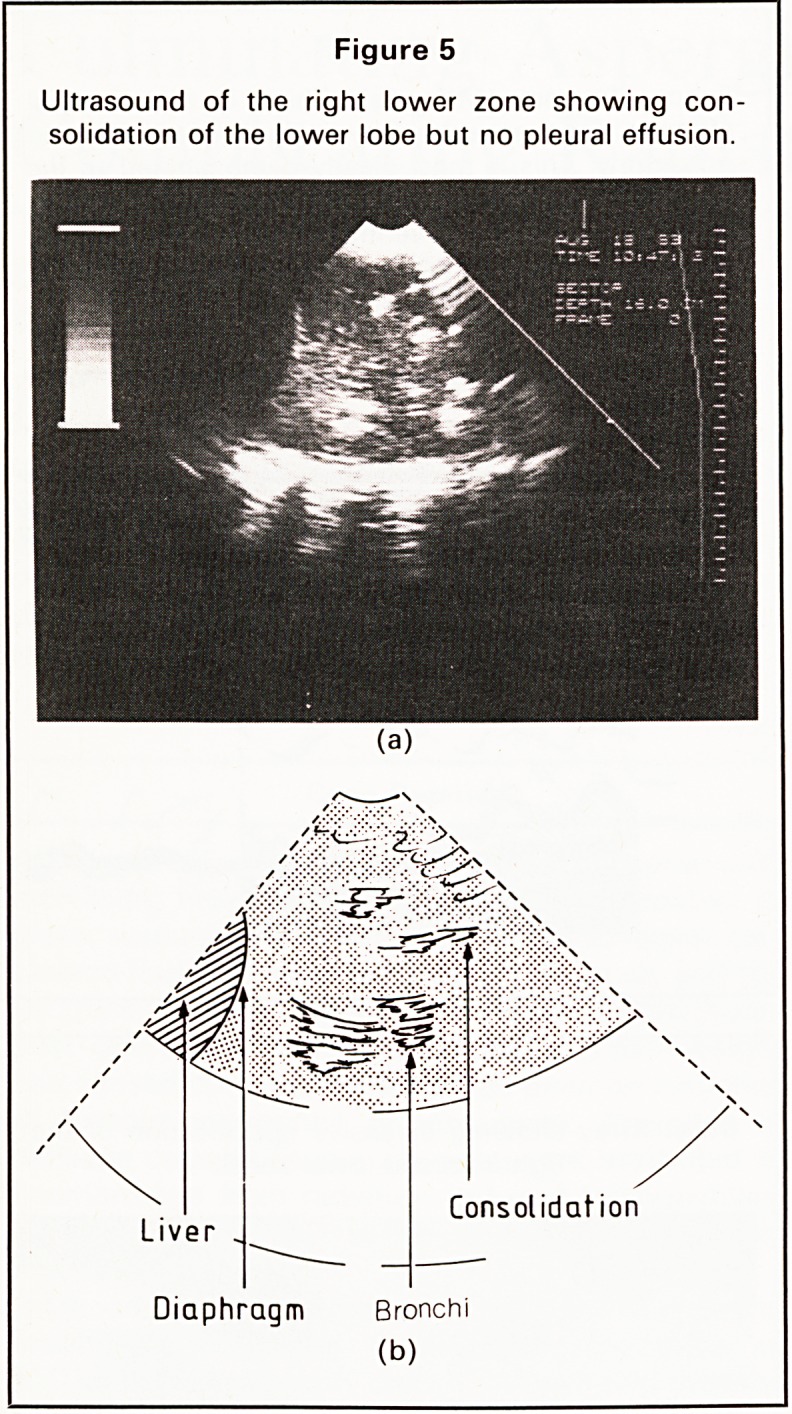# Fulminating Aspergillus Pneumonia Complicating Radiation Fibrosis

**Published:** 1984-07

**Authors:** D. W. Pitcher, P. Wood, P. R. Goddard, J. R. Rees

**Affiliations:** Bristol Royal Infirmary; Bristol Royal Infirmary; Bristol Royal Infirmary; Bristol Royal Infirmary


### Fulminating Aspergillus Pneumonia Complicating Radiation Fibrosis

**D. W. Pitcher, P. Wood, P. R. Goddard, J. R. Rees**
Bristol Royal Infirmary

Invasive pulmonary Aspergillosis is an unusual opportunist infection. This report describes a patient with fulminating Aspergillus pneumonia in a region of radiation damage consequent on therapy for an oesophageal carcinoma. Computed tomography and ultrasound techniques were useful in the diagnosis and management.

**Figure 1**

Chest X-ray showing an enlarged heart shadow and opacification in the right lung field.

### Case Report

A 39 year old woman was admitted to hospital with increasing breathlessness and cough productive of green sputum over 3 months. An oesophageal carcinoma had been resected 3 years previously and she had received prophylactic radiotherapy to the thorax post-operatively. At follow-up failure to gain weight had caused concern but there had been no evidence of tumour recurrence or metastasis. Episodes of melaena causing severe anaemia were attributed to haemorrhage from radiation-induced telangectasia, seen at endoscopy in the intrathoracic gastric mucosa.

On admission the patient was thin, febrile and breathless. She had a regular tachycardia of 120/minute. Inspiratory crackles were heard over the right upper lobe. The remainder of the examination was normal. Chest radiograph showed an enlarged heart shadow and opacification in the right lung field, particularly in the right upper zone (Figure 1). Computerised tomography showed a large cavitating lesion in the apex of the right lung with pleural thickening (Figure 2). There was patchy opacification within the right upper and middle lobes. The appearances were those of a destructive pneumonia in an area of radiation fibrosis. Echocardiography showed the apparent cardiomegaly to be due to a pericardial effusion (Figure 3).

Diagnostic possibilities included tumour recurrence, aspergillosis, tuberculosis and pyogenic infection. Initial sputum culture yielded no organisms that typically cause cavitation and previous treatment with oral amoxycillin had been ineffective. Additional antibiotic treatment with oral cotrimoxazole was initiated and subsequently intravenous cefuroxime and oral metronidazole were given, without clnical improvement. Acid fast bacilli were not found in the sputum and a tuberculin test was negative. Extensive investigation, including sputum cytology, upper gastrointestinal endoscopy and biopsy, and pericardial aspiration, failed to demonstrate evidence of malignancy. Thin barium swallow showed no evidence of tracheo-oesophageal fistula, or other cause for aspiration into the lung. Aspergillus pneumonia was supported by the finding on the blood film of a modest eosinophilia ($0.84\times109/1$) and confirmed by high titres of circulating precipitins to Aspergillus (1/32, rising to 1/64 and 1/128). Aspergillus fumigatus was subsequently isolated from the sputum.

Treatment was commenced with daily intravenous infusions of 40 mg amphotericin after an initial test dose of 10 mg. Although the patient tolerated this

**Figure 2**

Computed tomogram of the lung apices. In (a) the grey scale has been set to show soft tissues and bone. The same scan is shown in (b) but the settings have been altered to show the pulmonary vasculature. There is a large region of cavitation in the apex of the right lung with surrounding pleural thickening.

(a)

(b)

**Figure 3**

Echocardiogram (M-mode). There is a pericardial effusion seen as echo-free regions anteriorly and posteriorly. This is most clearly demonstrated to the right of the echocardiogram. (AW=anterior right ventricular wall. RV=right ventricle. IVS=interventricular septum. LV=left ventricle. PW=posterior left ventricular wall. AML=anterior mitral leaflet.)

**Figure 4**

Chest X-ray showing extensive opacification of the right mid and lower zones.

treatment well her fever persisted and serial radiographs showed increasing shadowing in the upper and mid zones of the right lung. Fibreoptic bronchoscopy showed no evidence of bronchial obstruction. Corticosteroid therapy was introduced with marked clearing of pulmonary opacification and resolution of fever. Erythromycin was given to cover the remote possibility of additional opportunist infection such as Legionella (serology was negative). After a brief improvement she became more breathless. Chest radiograph showed extensive opacification of the right mid and lower zones (Figure 4). Pleural aspiration was attempted to exclude an empyema, but yielded no fluid. Ultrasound examination of the lung showed pulmonary consolidation but no intrapleural fluid (Figure 5).

Despite continued treatment the patient deteriorated further and died 2 months after admission. Autopsy confirmed a destructive pneumonia in the right lung and a fibrinous pericarditis. There was no

**Figure 5**

Ultrasound of the right lower zone showing consolidation of the lower lobe but no pleural effusion.

(a)

(b)

evidence of recurrent or metastatic carcinoma. In the intrathoracic portion of the stomach there were 2 large ulcers, one of which had eroded into the right main bronchus creating a fistula.

### DISCUSSION

Fungal pneumonias are rare and usually occur as opportunist infections in immunocompromised patients. Aspergillus pneumonia is well recognised in this context[1,2] but has occurred relatively infrequently in some series.[3,4] The present case is unusual in that the infection developed in a lung damaged by radiation, in the absence of residual neoplasia or immunosuppressant therapy. An underlying immune deficiency cannot be excluded but there had been no history of previous skin or mucosal infections to suggest a longstanding susceptibility to fungal infection. Radiotherapy probably contributed to the development of the infection by causing parenchymal fibrosis and possibly bronchiectasis, with accumulation of secretions allowing growth of the fungus. Two cases of aspergilloma complicating radiotherapy have been reported by Ward and Davies[5] who found reports of 2 additional cases.

Invasive aspergillosis has not been reported previously as a complication of radiotherapy, and the role of local radiation-induced impairment of immunity in the genesis of this pneumonia remains speculative. An element of undernutrition may have increased susceptibility to opportunist infection, and once this was established the use of several broad spectrum antibiotics may have encouraged growth of the fungus. The progressive nature of this illness despite apparently adequate anti-fungal therapy is a disappointing reminder of the high mortality from Aspergillus pneumonia.[1] Nevertheless successful treatment of this infection in some patients[6,7] underlines the importance of early diagnosis and treatment.

Diagnosis may be difficult and should not depend on the isolation of the fungus which may take some days. As in our patient, eosinophilia may be a valuable clue and high titres of circulating Aspergillus precipitins are the best hope of an early diagnosis. These investigations were performed in our patient after pulmonary cavitation was detected by computed tomography. Subsequent review of the chest radiographs showed that the pulmonary changes had developed slowly, but the cavitation had been masked by dense pleural thickening. Cavitation was readily visible on the cross-sectional image.

Other imaging techniques were also valuable aids to management. Apparent cardiac enlargement had initially raised a suspicion of left heart failure contributing to the patient's breathlessness. Echocardiography showed the pericardial effusion, permitting timely aspiration. Real-time ultrasound was also used to distinguish between pleural effusion and basal pulmonary consolidation; thus repeated fruitless attempts at pleural aspiration were avoided. This technique should be considered in any case where an initial attempt at pleural aspiration fails. It may show that there is no pleural effusion, or permit successful aspiration by identifying the position of a loculated effusion. Thus this case illustrates some of the additional imaging techniques which may clarify the nature of non-specific abnormaltities on a plain chest radiograph.

The gastric ulcers and bronchial fistula found at autopsy were not identified by contrast radiography or endoscopy. It seems improbable that these large ulcers would have been missed by both techniques. We believe that the ulceration occurred late in the patient's illness and that subsequent fistula formation resulted in her further deterioration due to aspiration into the right lower lobe. The case also illustrates some potential hazards of radiotherapy, the risks of which must be balanced against the risk of tumour recurrence when this form of treatment is used prophylactically.

### ACKNOWLEDGEMENTS

The authors are grateful for the assistance of Dr. P. Wilde, Dr. G. Laszlo, Miss J. Hugh, Mr. E. Turnbull and the Department of Medical Illustration.

### REFERENCES

1. BODEY, G. P. (1966) Fungal infections complicating acute leukaemia. *J. Chronic. Dis.* 667–687.
2. MEYER, R. D., YOUNG, L. S., ARMSTRONG, D., YU, B. (1973) Aspergillosis complicating neoplastic disease. *Am. J. Med.* **54**, 6–15.
3. SINGER, C., ARMSTRONG, D., ROSEN, P. P., WALZER, P. D., YU, B. (1979) Diffuse pulmonary infiltrates in immunosuppressed patients. Prospective study of 80 cases. *Am. J. Med.* **66**, 110–120.
4. JAFFE, J. P., MAKI, D. G. (1981) Lung biopsy in immunocompromised patients: one institution's experience and an approach to management of pulmonary disease in the compromised host. *Cancer* **48**, 1144–1153.
5. WARD, M. J., DAVIES, D. (1982) Pulmonary aspergilloma after radiation therapy. *Br. J. Dis. Chest* 76: 361–364.
6. DALY, B. D. (1983) Successful treatment of invasive pulmonary aspergillosis in a patient with acute leukaemia. *Irish J. Med. Sci.* **152**, 103–105.
7. McLEOD, D. T., MILNE, L. J. R., SEATON, A. (1982) Successful treatment of invasive pulmonary aspergillosis complicating influenza A. *Br. Med. J.* **285**, 1166–1167.

## Figures and Tables

**Figure 1 f1:**
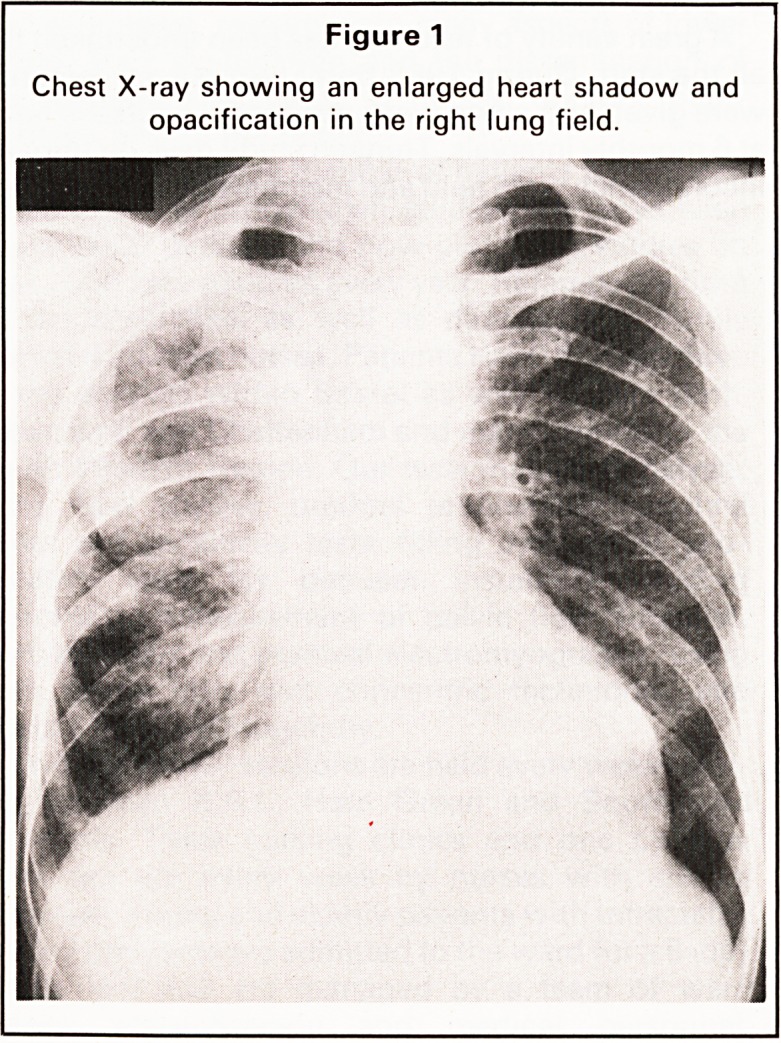


**Figure 2 f2:**
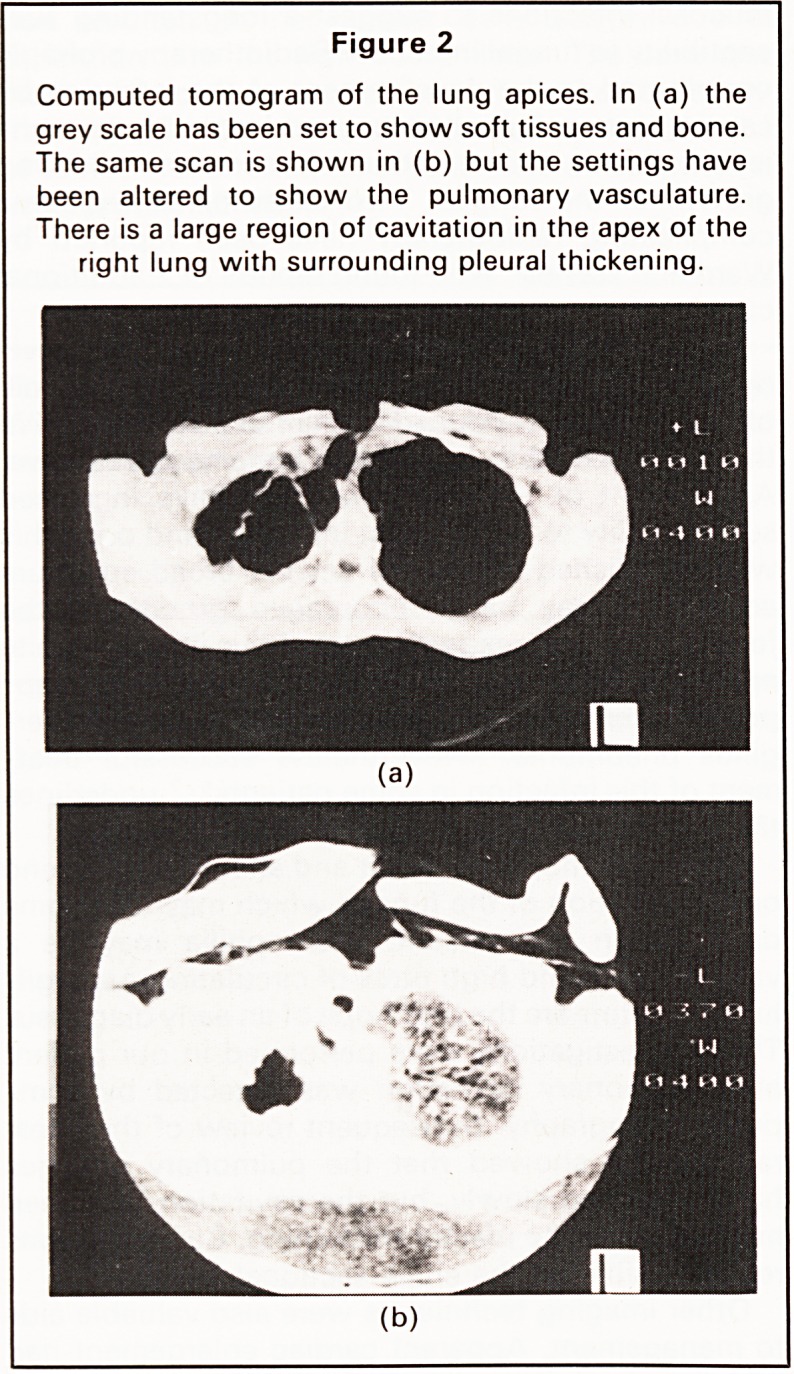


**Figure 3 f3:**
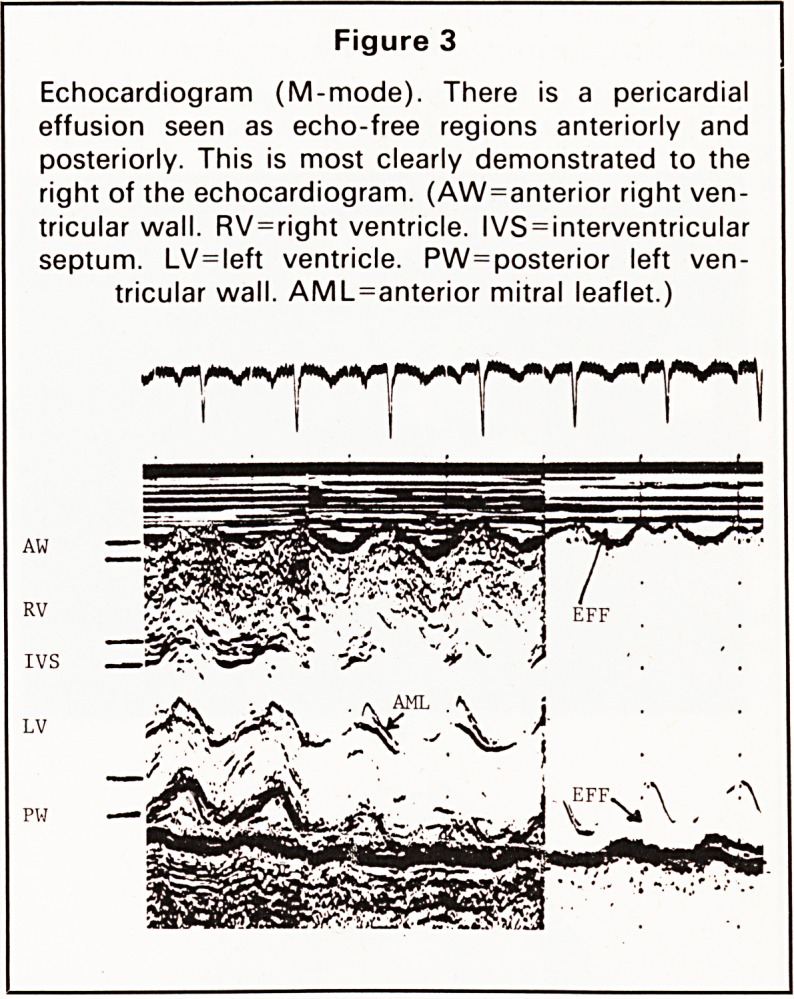


**Figure 4 f4:**
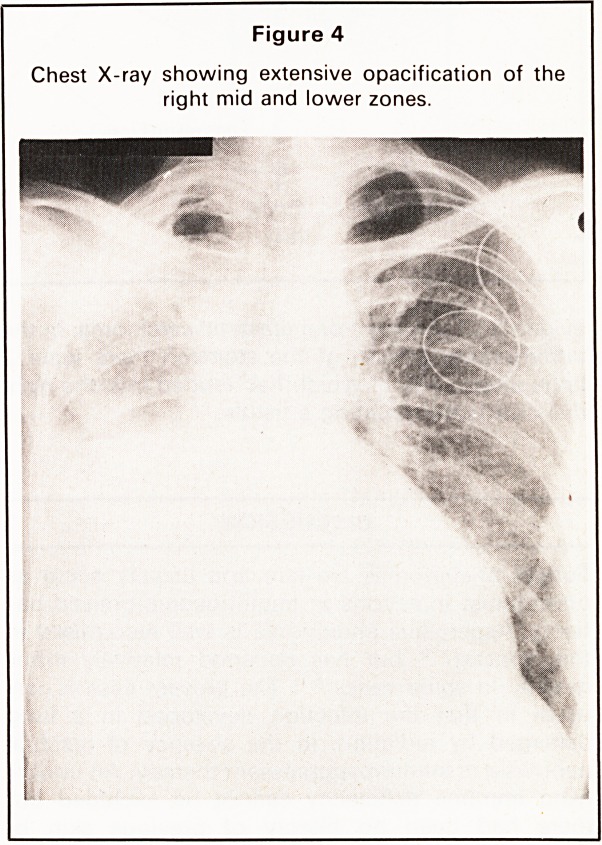


**Figure 5 f5:**